# Effects of Performing Eccentric Contractions to Failure After Concentric Muscle Failure in Resistance Training Sessions: Protocol for a Within-Participant Randomized Trial

**DOI:** 10.2196/67537

**Published:** 2026-01-08

**Authors:** Pedro Henrique Alves Campos, Renan Vieira Barreto, Gabriel Fontanetti, Leonardo Santos Lopes da Silva, Matheus Machado Gomes, Leonardo Lima

**Affiliations:** 1 School of Physical Education and Sport of Ribeirão Preto University of São Paulo Ribeirão Preto Brazil; 2 Department of Physical Education Institute of Biosciences São Paulo State University Rio Claro Brazil; 3 Department of Internal Medicine Ribeirão Preto Medical School University of São Paulo Ribeirão Preto Brazil

**Keywords:** eccentric exercise, resistance training, muscle hypertrophy, muscle function, exercise-induced muscle damage

## Abstract

**Background:**

Resistance training is a well-established strategy to promote muscle hypertrophy and strength gains. Performing sets to concentric muscle failure (MF_CON_) is commonly used to maximize neuromuscular adaptations. However, after reaching MF_CON_, there is a remaining capacity for eccentric contractions that could be used. Increasing eccentric contraction volume may represent a promising and practical alternative to enhance training volume load and optimize adaptations, although its effectiveness in this specific application has not yet been tested.

**Objective:**

This study aims to investigate whether performing additional eccentric contractions to eccentric muscle failure (MF_EXC_), after the occurrence of MF_CON_, enhances neuromuscular and morphological adaptations beyond those promoted by a traditional protocol to MF_CON_.

**Methods:**

In a randomized within-subject design, untrained young adult females will perform 2 upper-limb resistance training protocols over 10 weeks, including traditional (TRAD) training to MF_CON_ and a training protocol (ECC+) consisting of sets to MF_CON_ followed by eccentric-only contractions to MF_EXC_. Each arm will be assigned to one of the protocols. Sessions (twice per week) will consist of 6 sets of unilateral elbow flexion with a load between 9 and 12 repetition maximum, with 2-minute rest intervals. Muscle function (isometric, concentric, and eccentric strength) and body composition (biceps brachii and brachialis muscle thickness and dual-energy x-ray absorptiometry [DXA]–based analysis) will be assessed pre and post intervention. Comparisons between limbs and across time will be analyzed using 2-way ANOVA. The level of significance will be set at *P*<.05.

**Results:**

As of July 2024, a total of 7 participants have completed the intervention. Data collection was conducted between March and July 2024, with a new phase planned for the first half of 2025. Manuscript submission is expected in the second half of 2025.

**Conclusions:**

If the hypothesis is confirmed, the ECC+ protocol may represent a practical, simple, and low-cost strategy to increase training volume and optimize strength and hypertrophy outcomes. This study may contribute to evidence-based resistance training prescriptions, particularly for women, and support the use of additional eccentric contractions as an effective tool to enhance localized muscle adaptations.

**International Registered Report Identifier (IRRID):**

DERR1-10.2196/67537

## Introduction

### Background

Resistance training (RT) with free weights and gym machines is widely accepted as an effective intervention for inducing increases in strength and muscle size (ie, muscle hypertrophy) [1]. Evidence indicates that manipulating RT variables, such as volume (ie, number of sets) and intensity (ie, percentage of one-repetition maximum [RM]), is crucial for optimizing these adaptive responses [2-7]. The improvement in muscle strength in response to RT [1,5,8,9] primarily occurs due to neuromuscular and physiological adaptations that are important for muscle health, such as improved recruitment of motor units during contraction [10,11] and increased coordination resulting from enhanced activation of agonist muscles and reflex inhibition of antagonist muscles, contributing to more precise, coordinated, and efficient movements [12-14]. Additionally, RT allows for improvements in muscle capillary density and vascularization (ie, angiogenesis), which enhances the delivery of oxygen and nutrients to muscle fibers, resulting in greater metabolic efficiency and reduced fatigue during exercise [15]. Increases in strength in response to RT can be measured in various ways, such as the determination of 1RM increases for a given exercise [5], increases in maximal concentric, eccentric, and isometric torque production by the trained muscles, and changes in the optimal angle for force production [16-18], which can be assessed through specific tests on isokinetic equipment [19].

Among the variables that can be manipulated to enhance the effectiveness of RT, one particularly noteworthy strategy is performing sets to concentric muscle failure (MF_CON_), which has been shown to effectively promote increases in muscle cross-sectional area [20-22]. This approach ensures that the individual completes the maximum number of repetitions possible for a given load. Muscle failure is defined as the point at which an individual can no longer complete a repetition with proper technique, even when exerting maximal effort. Concentric failure occurs when the individual is no longer able to perform the shortening phase of the muscle, for example, during a biceps curl, it is the moment when the person can no longer lift the weight. Taking the set to MF_CON_ can result in the recruitment of a higher number of motor units, especially high-threshold motor units and muscle fibers [10], which are not normally activated during less intense efforts or those that do not bring the muscle close to MF_CON_. This can result in greater activation and stress on muscle fibers. Moreover, at lower intensities, a greater number of repetitions is required to reach MF_CON_. In these cases, due to the higher number of repetitions and the high degree of muscle stress, there is greater accumulation of metabolites, such as lactate, hydrogen ions, and inorganic phosphate in muscle cells, leading to muscle swelling, which can also be a trigger for hypertrophy [23].

Another benefit of performing sets close to MF_CON_ is the greater accumulation of time under tension, an important variable for the hypertrophic process due to its role in optimizing mechanotransduction [24]. Although recent evidence shows that reaching MF_CON_ is not a sine qua non condition for muscle hypertrophy [4,25-27], it is well established that protocols incorporating this strategy are effective in promoting muscle mass increase [4,6,21,28].

Equally important, the amount of mechanical work performed in an RT session, typically estimated by the product of resistance (kg) divided by the number of repetitions performed (also referred to as volume load [VL]), is considered one of the most important variables for developing muscle strength and, especially, hypertrophy [6,29]. Therefore, progressive increases in the resistance used or the number of repetitions performed—ideally close to MF_CON_—in a given exercise are recommended during RT interventions to promote an increase in VL [1,30,31].

There is a large body of research investigating different strategies for manipulating RT variables, especially volume and intensity, and comparing their impacts on hypertrophic responses and improvements in muscle function after chronic interventions. Examples include pyramid training systems [32], drop-set [33], rest-pause [34,35], cluster set [36], and conjugate sets [37-39], among others. However, it is commonly observed that in most studies comparing so-called advanced techniques or training models with the traditional method (ie, concentric failure), regardless of how the factors (ie, volume and intensity) are manipulated, when the VL is kept equal, the responses tend to be similar. Therefore what seems to determine the adaptations to RT is not the technique itself, but its ability to alter the final product, that is, VL [5]. Such techniques should be studied, taking into account their main practical application, increasing the training VL in order to verify whether such an increase would result in a better response. A plausible VL-increasing strategy that has yet to be investigated is performing sets of repetitions to eccentric muscle failure (MF_EXC_). In the context of resistance training, it is common to distinguish between 2 main types of muscle failure, such as concentric and eccentric. Eccentric failure refers to the inability to control the muscle lengthening phase under load, such as during the lowering portion of a biceps curl. In this case, even with external assistance to initiate the repetition (thus overcoming the concentric phase), the individual reaches such a high level of fatigue that they can no longer resist the load during the return phase, causing the weight to drop uncontrollably. Therefore, while concentric failure represents the limit of the ability to generate force to move a load, eccentric failure indicates the exhaustion of the capacity to control that load during the muscle’s lengthening phase.

Each repetition in RT consists of a concentric phase (when the external resistance is moved as a result of muscle shortening) and an eccentric phase (when the movement of the external resistance is controlled by active muscle lengthening) [40,41]. The distinct mechanisms of force production during eccentric and concentric contractions result in significant differences in force and power generation, energy cost, and fatigue between the 2 types of contraction [41-44].

For example, a recent meta-analysis estimated that maximal eccentric strength is approximately 40% greater than maximal concentric strength [40]. More relevant to this study, it was demonstrated that to achieve the same level of fatigue observed in a concentric exercise (ie, 40% reduction in maximal isometric force), a 28.8% greater volume of contractions (ie, 134 vs 104) was required when the exercise was performed eccentrically [42]. Supporting this, it has also been shown that when the same external resistances are used (ie, 70%-95% of 1RM), protocols of concentric and eccentric contractions to MF_CON_ and MF_EXC_ result in a substantially greater volume (64% vs 152%) of the latter type of contraction compared to the former [44]. Based on this information, it can be assumed that (1) during traditional RT sessions (performed with free weights and gym machines), the eccentric phase of repetitions is performed at a relatively lower intensity (ie, % of maximal eccentric strength) since the determination of external resistance is based on the mass the performer can move during muscle shortening (ie, concentric phase); and (2) in sets performed to MF_CON_, MF_EXC_ is not reached, resulting in a reserve of eccentric contractions that could contribute to an increase in the VL of the session but is underused.

In addition to having distinct intrinsic characteristics from concentric contractions, eccentric contractions, when emphasized in specific RT protocols, also seem to induce unique adaptations compared to traditional RT protocols. For example, when using eccentric loads greater than maximal concentric strength (ie, >1RM), exclusively eccentric training appears to promote greater overall strength gains (ie, combined concentric, isometric, and eccentric strength) compared to exclusively concentric and traditional training (ie, alternating concentric and eccentric contractions) [45]. Furthermore, high-frequency eccentric training (2.5 repetitions per second) performed on an isokinetic ergometer was associated with greater increases in muscle power (assessed through changes in jump performance) compared with traditional training [46].

An interesting characteristic of eccentric training is that it appears to promote regional hypertrophy, with a tendency to induce greater increases in the cross-sectional area of the distal portion of the vastus lateralis [47,48]. There is also a preferential increase in type II muscle fiber size, as well as an increase in cross-sectional area and tendon stiffness induced by training with rapid, high-intensity eccentric contractions. Furthermore, from a muscle mechanics perspective, training with an emphasis on eccentric contractions has been shown to result in optimization of the stretch-shortening cycle, allowing for improvements in muscle power and inducing an increase in muscle fascicle length (ie, addition of sarcomeres in series), which may lead to increased muscle shortening velocity and force production when the muscle is in a stretched state [49]. Furthermore, when comparing the chronic effects of training with different types of contractions of the elbow flexor muscles, where one group performed only concentric contractions, another performed concentric and eccentric contractions, and a third group performed only eccentric contractions, similar increases in strength and muscle mass were found between the eccentric and concentric–eccentric groups, despite a lower total training volume for the first [50]. These findings indicate a greater contribution of this type of contraction to muscular adaptations.

Eccentric training at different speeds also seems to influence cross-education (ie, increased contralateral eccentric strength) [51]. It has also been demonstrated that unilateral eccentric training promotes attenuation of muscle function loss in the contralateral limb induced by immobilization [52]. This cross-education effect is not exclusive to eccentric training; however, the cited studies demonstrate that the effect is more pronounced with eccentric training compared to traditional training. It is essential to note that investigations identifying contralateral transfer of neuromuscular adaptations induced by eccentric training were conducted with one limb exercising while the contralateral limb (which benefited from cross-education) remained inactive.

### Justification

Considering that (1) the VL of RT sessions is positively associated with the magnitude of functional and morphological adaptations induced in skeletal muscle after a long-term RT intervention; (2) traditional RT protocols performed to MF_CON_ do not use a reserve of eccentric contractions that still exists after this phenomenon; (3) when MF_CON_ occurs, skeletal muscle is in an anabolic state due to both the VL and the metabolic state induced by anaerobic metabolism activation; and (4) different RT strategies with some emphasis on eccentric contractions result in unique functional and morphological adaptations in skeletal muscle. There is a gap in the literature regarding the impacts of increasing VL during RT sessions by performing eccentric muscle contractions to MF_EXC_ after the occurrence of MF_CON_, a situation where hypertrophic signaling in the muscle is already increased. Given the rationale developed above, it is reasonable to consider that increasing VL using this strategy may result in significantly greater increases in both cross-sectional area and muscle function. If confirmed, the proposed protocol could be efficiently and easily implemented in the context of physical education and sports professionals when the goal is to promote increases in muscle mass and function.

### Objectives

The objectives of this study are (1) to compare, through a randomized intraparticipant design, the acute and chronic impacts of using sets to MF_EXC_ with using sets to MF_CON_ on the VL produced during training sessions; (2) to compare the chronic impacts of using sets to MF_EXC_ with using sets to MF_CON_ on muscle morphological variables (body composition and muscle cross-sectional area); and (3) to investigate the chronic impacts of applying the 2 mentioned techniques on muscle function (maximal isometric strength, rate of development of isometric strength, maximal concentric strength, and maximal eccentric strength).

### Hypotheses

The hypotheses of this study are associated with the specific objectives presented above and are as follows: (1) the total work produced during training sessions will be greater for the intervention involving sets to MF_EXC_; (2) the magnitude of changes in muscle morphological variables will be greater after the intervention involving sets to MF_EXC_; and (3) improvements in muscle function will be more pronounced after the intervention involving sets to MF_EXC_.

## Methods

### Participants

Young adult females (18-30 years old) will be invited to participate in the study. As an inclusion criterion, participants must have no recent experience (ie, 6 months) with upper body strength training of any kind (ie, resistance training, functional training, Pilates, and CrossFit). Exclusion criteria will include a history of musculoskeletal or joint injuries in the upper limbs within the past 12 months and use of anabolic steroids. Participants will be instructed to maintain their usual dietary habits and regular physical activity routines.

### Experimental Design

The study will be conducted as a within-participant randomized trial. Each participant will undergo 2 different interventions over 10 weeks, each being performed on one of their upper limbs, with 2 training sessions per week separated by a minimum of 48 hours and a maximum of 96 hours. The intervention for each participant’s upper limb will be randomly assigned via electronic randomization. Using commonly available online randomization tools, the first randomization draw will determine whether the left or right upper limb will be selected. Next, the intervention to be applied to the previously selected limb (traditional or ECC+) will be randomly selected. Finally, a randomization draw will decide whether the previously selected protocol and limb will be conducted before or after the unselected protocol (ie, both protocols will be performed in the same session). No counterbalancing based on limb dominance (ie, right-handedness) will be implemented in either the applied protocol or the order of performance. The limb assigned to traditional training (TRAD) will follow a protocol with sets of repetitions until concentric failure (MF_CON_). The limb assigned to the eccentric overload training protocol (ECC+) will follow the same exercise protocol, but with the addition of exclusively eccentric contractions until eccentric failure (MF_EXC_) after reaching MF_CON_.

The existence of a widely reported cross-education effect, particularly for variables related to muscle function, can be considered a limitation of this design. However, there is a growing trend in strength training research suggesting that, despite this effect, experimental designs comparing active unilateral strength training interventions (ie, with the control limb remaining nonsedentary) performed on each limb of the same individual are more suitable than designs comparing interventions in 2 different groups of people, even if randomized [53]. This is justified by the greater control in such experimental designs over intrinsic factors (eg, gene expression of protein transcription factors, muscle fiber type distribution, and capillary-to-fiber ratio) and extrinsic factors (eg, sleep quality, training level, and diet), which are known to influence responses to strength training and cannot be controlled in designs involving different individuals in groups. Furthermore, when the primary outcome of the investigation is muscle hypertrophy, the unilateral training design appears even more suitable, as there is evidence that there is no cross-education effect for this type of outcome [54-56].

**Figure 1:**
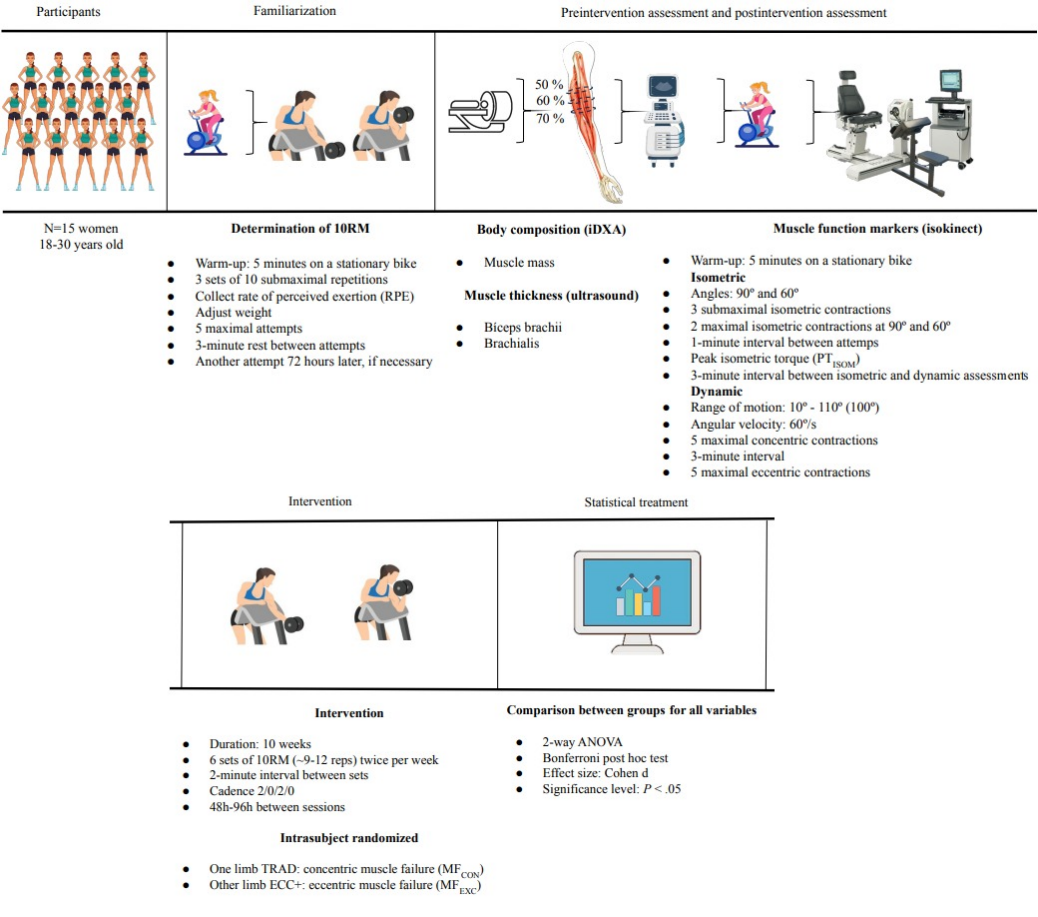
Timeline of the experiment including procedures and assessments. RM: repetition maximum.

Neuromuscular function and body composition markers will be collected 1 week before and 1 week after the interventions and compared between limbs. The VL performed in each training session and the session’s perceived effort will be recorded for comparison over time and between limbs. The experimental design of the study is illustrated in Figure 1.

### Training Protocol

**Figure 2:**
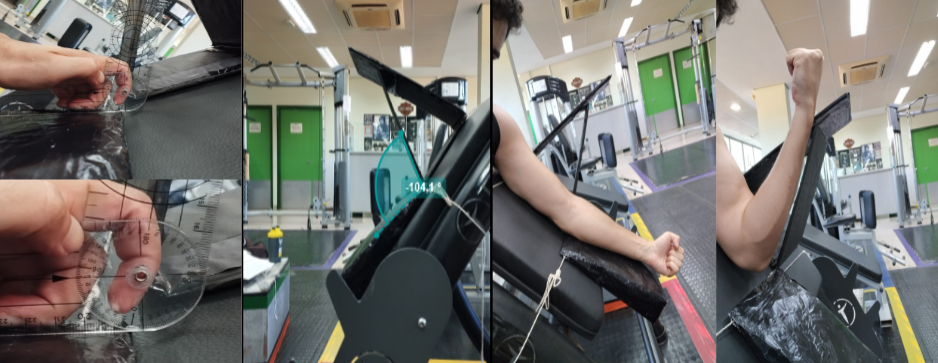
Illustration of the settings adopted for the training sessions.

The exercise to be performed by participants in this study will be unilateral elbow flexion using a preacher curl bench, Scott, OriGym Brasil, with 45º shoulder flexion, carried out at the Laboratory of Kinanthropometry and Human Performance of the School of Physical Education and Sports of Ribeirão Preto. For both protocols, participants will be instructed to perform repetitions at a cadence of 2/0/2/0 seconds (ie, 2 seconds for the concentric phase, 0 seconds of isometry between the concentric and eccentric phases, 2 seconds for the eccentric phase, and 0 seconds of isometry between the eccentric and concentric phases) with the assistance of the Beats Metronome app developed by Stonekick Apps, which will set the rhythm to 60 bpm (ie, one beat per second). This method will help control the cadence, as there will be an auditory reference at the beginning, middle, and end of each contraction. Muscle failures in the concentric and eccentric phases will be considered as the first concentric and eccentric repetitions, respectively, that the participant cannot complete within a 2-second interval. The range of motion adopted will be 100º, limited by a physical barrier during extension (maintaining a slight flexion of 10º at the end of extension and a visual apparatus for reference at the end of flexion, where the participant’s forearm should be parallel to the apparatus at the end of the range of motion of the concentric phase and beginning of the eccentric phase), as illustrated in Figure 2.

### Initial Load and Training Protocol

The initial load adopted will be 10RM, which should be maintained throughout the training in the range of 9-12 maximum concentric repetitions. This means that whenever a participant reaches MF_CON_ with more than 12 or fewer than 9 repetitions in a set, the load will be increased or decreased, respectively, in the subsequent set. Load adjustments will be made in increments of at least 1 kg. For the ECC+ protocol, additional eccentric contractions will be performed with the same load used during repetitions until MF_EXC_.

Training sessions will consist of 6 sets of elbow flexions with 2-minute recovery intervals. The recovery interval will begin when the participant reaches MF_CON_ or MF_EXC_ (for TRAD and ECC+ protocols, respectively). To allow for additional eccentric contractions after MF_CON_ in the ECC+ protocol, examiners will move the weight to the elbow-flexed position, where participants will start the eccentric phase. This will make the concentric phase of elbow flexion load-free for the participants—except for the weight of the arm itself, which will need to be moved to the flexed position.

### Determination of 10RM Load

The determination of the 10RM load for the elbow flexion exercise will be conducted following a warm-up protocol consisting of 3 sets of 10 submaximal unilateral elbow flexions with a 3 kg load on the preacher curl bench, with a 3-minute rest between sets. At the end of the 30 submaximal warm-up contractions, participants will be asked to register their rate of perceived exertion (RPE) using the OMNI scale [57].

The load for the first attempt will be based on the RPE during the warm-up with a 3 kg load. If the reported value is between 8 and 9, the load for the first attempt will be increased by 0.5 kg. If the reported value is between 5 and 7, the load will be increased by 1-2 kg. If the reported value is less than 5, the initial load will be increased by 2.5-3 kg.

All attempts to determine the 10RM load will follow the same protocol described for the training sessions (ie, metronome-guided rhythm, 2/0/2/0 seconds cadence, and 100° range of motion). A maximum of 5 attempts per session will be allowed, with 3-minute intervals between attempts. If participants reach MF_CON_ before the 10th repetition during an attempt, the load for the next attempt will be decreased by 1-2 kg, depending on the number of repetitions required to reach the 10th. In cases where MF_CON_ does not occur by the 10th repetition, and participants successfully complete the 11th repetition, the attempt will be terminated, and the load for the next attempt will be increased by 0.5-1 kg. If the 10RM load is not successfully determined within the 5 attempts made in a single day, the session will be concluded, and a new determination session will be scheduled for another day, allowing at least 72 hours of recovery.

### Monitoring of VL

VL will be quantified as the total work performed in each session. Perceived exertion (RPE) will also be considered as a perceptual indicator of internal training load. VL for each session will be calculated as the product of resistance (kg) and the number of repetitions completed. For the ECC+ protocol, additional eccentric contractions will be multiplied by 0.5, as they represent half of the concentric–eccentric contraction cycle. Incomplete contractions that characterize the points of concentric failure (MF_CON_) and eccentric failure (MF_EXC_) will not be included in the calculations.

The RPE will be reported by the participants immediately after each set using the OMNI scale [57]. Additionally, the overall session RPE will be assessed 15 minutes after the end of the session using the same scale, with instructions for participants to evaluate the entire session, not just the last set performed.

### Markers of Neuromuscular Adaptation to Training Protocols

Body composition of both arms will be determined using dual-energy x-ray absorptiometry (DXA; Lunar, iDXA, GE Healthcare) in a supine position. The variable of interest for this study will be the muscle mass of the arm and the entire upper limb of the participants.

The thickness of the biceps brachii and brachialis muscles will be determined at 3 locations, based on percentages of the distance between the posterior margin of the acromion of the scapula and the olecranon of the ulna, namely proximal (50%), middle (60%), and distal (70%) [58], using images obtained by a mode-B ultrasound device (Saevo FP 102, Ribeirão Preto) with a 40 mm linear transducer and a frequency of 10 MHz. Participants will remain in a supine position for 5 minutes before recordings to allow for fluid accommodation. The transducer will be positioned perpendicularly to the skin over the muscle and transversely to the limb. Three images of each assessed region of both muscles will be captured and exported. The ImageJ software (National Institutes of Health) will be used to determine the thickness of each muscle by calculating the difference between the superficial and deep fascias, as well as the total muscle compartment thickness, by calculating the difference between the superficial fascia of the muscle compartment and the osteomuscular interface. Reliability of ultrasound measurements will be carried out by means of the intraclass correlation coefficient.

Muscle function will be evaluated using an isokinetic dynamometer (System 4, Biodex Systems) with participants positioned on a preacher curl bench set at a 45º angle adjusted to the dynamometer. Peak isometric torque and maximum rate of torque development (RTD) will be determined from 2 maximal voluntary isometric contractions of 5 seconds at 2 different angles, 60º and 90º of elbow flexion, with a 1-minute interval between each attempt. After proper positioning on the dynamometer, following the manufacturer’s instructions, and a standardized warm-up (5 minutes of exercise with a self-selected load on a stationary bike followed by 3 submaximal isometric contractions), participants will be instructed to flex the assessed elbow as quickly and with as much force as possible, sustaining the maximal contraction for 5 seconds. A 1-minute rest will be observed between the 2 contractions at each angle. The torque-time curves will be filtered and analyzed in MatLab (Mathworks, IBM), with the highest torque value recorded in the 2 contractions considered as peak isometric torque and the steepest slope of the torque-time curve as RTD. These procedures will be performed for each investigated angle (ie, 60º and 90º of elbow flexion). The contraction with the highest torque value will be used for analysis. Peak torque will be calculated as the highest value on the torque-time curve. The maximum RTD will be calculated as the steepest slope of the torque-time curve. The onset of muscle contraction will be defined as the point where torque values exceed 2.5% of the difference between baseline and peak torque values [59]. For more details on the procedures for filtering and analyzing isometric torque data, refer to [60]. Three minutes after the assessment of isometric strength, dynamic muscle function will be evaluated using nonintercalated maximal isokinetic concentric and eccentric contractions. First, 5 maximal isokinetic concentric contractions of the elbow flexors will be performed with a range of motion of 100° (10º-110º of elbow flexion) at an angular velocity of 60°/s. This range of motion was chosen to allow for greater joint comfort during movement, avoiding full extension at 0º at the beginning of the movement. Additionally, a semipronated grip at 30º is standardized for greater wrist joint comfort. The interval between contractions will be approximately 1.5 seconds, while the dynamometer arm returns passively to the initial position. After a 3-minute recovery interval, 5 maximal isokinetic eccentric contractions of the elbow flexors will be performed with the same range of motion (100°: 10º-110º), angular velocity (–60°/s), and interval between contractions. The highest torque values produced for each contraction will be recorded, and the average of the highest values from the 5 contractions (for each type of contraction) will be calculated and recorded as concentric torque peak and eccentric torque peak.

All data obtained from isokinetic force analyses and ultrasound muscle thickness measurements were performed by an evaluator who was blinded to which protocol each limb performed.

### Statistical Treatment

A formal a priori power analysis was conducted using G*Power software (version 3.1.9.7; Heinrich-Heine-University Düsseldorf). The calculation was based on an effect size of 0.57 [61], for a study that observed significant increases in elbow flexor muscle thickness following resistance training performed to failure, also over a 10-week period. Considering a power (1-β) of .80, a significance α level of .05, and a 2-tailed test for repeated measures (within-subject design), the estimated required sample size was 21 upper limbs. Accordingly, the minimum number of participants needed was 11, assuming each would complete both unilateral protocols. This estimation was based on a 10-week intervention period and informed the recruitment target of this study. The normality of data distribution will be assessed using the Shapiro-Wilk test. Nonparametric data will undergo a logarithmic transformation. The assumptions of homogeneity and sphericity of the data will be checked using Levene and Mauchly tests, respectively. Comparisons over time and between limbs for all dependent variables will be conducted using a 2-way mixed model ANOVA for repeated measures (time effect) and nonrepeated measures (limb effect). If significant effects of limb or time are identified, Bonferroni post hoc adjustments will be applied. Main effect sizes will be calculated and expressed as partial η². If significant interaction effects between limb and time are detected, pairwise comparisons between relevant points will be performed. Effect sizes for significant differences in dependent variables between limbs or over time will be calculated and expressed as Cohen *d*. The significance level adopted will be *P*<.05.

### Ethical Considerations

All participants will sign an informed consent form prior to their inclusion in the study, ensuring they are fully aware of their rights and of the possibility of withdrawing at any time without any repercussions. The data collected will be fully anonymized to ensure the privacy and confidentiality of the participants. No identifiable information will be retained, and robust protective measures will be implemented to safeguard all collected data. The present project was submitted on July 2, 2024, and approved by the Research Ethics Committee, with the Certificate of Presentation for Ethical Consideration: 76595923.8.0000.5659.

## Results

Up until now, a total of 7 participants have been recruited and completed the intervention. The first preintervention data collection was conducted on January 3, 2024, and the final postintervention test of the last participant was completed on October 7, 2024. Data collection from new participants is planned for the first semester of 2025. The paper is expected to be published in the second semester of 2025. This study was financed, in part, by the São Paulo Research Foundation (Fundação de Amparo à Pesquisa do Estado de São Paulo [FAPESP]), Brasil (process number 2023/13099-7). The workplan for the experiment is presented in Table 1.

**Table 1:** Baseline participant characteristics after matching.

Period	Month of execution								
	January 2024	February-March 2024	April-June 2024	July-August 2024	October-December 2024	January-March 2025	April-June 2025	July-September 2025	October-December 2025
Ethical assessment of the project (already completed)	✓								
Research team training (already completed)	✓	✓			✓				
Determining the reproducibility of assessments (already completed)	✓								
Data collection (ongoing)		✓	✓	✓	✓	✓	✓	✓	
Analysis of the data obtained					✓		✓	✓	
Discussion of the results obtained								✓	✓
Preparation of the final report								✓	✓
Preparation of abstracts and scientific articles									✓

## Discussion

### Anticipated Findings

The objectives of this study are to compare, through a randomized intraparticipant trial, the chronic effects of RT sets performed up to MF_EXC_, after MF_CON_, in relation to series performed only up to MF_CON_ on the VL produced during the training sessions, muscle mass, muscle thickness of the proximal, medial and distal regions of the arm, and muscle function assessed by maximum isometric, concentric, and eccentric strength. The hypotheses are that (1) the total work performed during the sessions will be greater in the sets performed up to MF_EXC_, (2) the muscle morphological adaptations will be more pronounced after the intervention with sets up to MF_EXC_, and (3) the improvements in muscle function will also be more pronounced with the ECC+ protocol. If the hypotheses that a greater training volume induces greater hypertrophy adaptations are confirmed, the results will align with previous findings in the literature [2,6,29,31]. If so, the proposed intervention could be implemented as a new practical method in the personal trainer’s professional context, providing session time efficiency (ie, increased training density), as the volume load could be substantially increased with sets taken to MF_EXC_, thereby inducing greater responses. Regarding regional hypertrophy, the analysis of the 3 regions (50%, 60%, and 70% of the arm length) may indicate that the thickness response is different between the studied protocols. Studies indicate that hypertrophy may occur nonuniformly throughout the muscle, being more pronounced in the regions that undergo greater stretching during the exercise [62]. In the preacher curl bench exercise, used in this study, peak torque occurs at the beginning of the concentric phase and at the end of the eccentric phase, which maximizes stress on the distal portion of the biceps brachii when stretching and suggests that exercise biomechanics plays a crucial role in regional hypertrophy [63]. Some possible limitations could be that the fatigue and muscle damage induced by the ECC+ protocol may not maximize hypertrophic responses, even with a higher volume load [64,65]. Another limitation is the choice to use this method on fusiform muscles, making it impossible to analyze pennation angle, which could be altered with greater emphasis on eccentric contractions, as well as not measuring fascicle length, which could be carried out in future work using the ECC+ method in other pennate muscles. The reason for choosing to investigate the elbow flexor muscles was exclusively related to the implementation logistics and the availability of equipment in our laboratory. However, as the study does not aim to answer questions related to kinesiological variables but rather to the manipulation of training variables, such as hypertrophy and strength, it is likely that this choice will not significantly interfere with the main outcome. Additionally, the unequal volume load between limbs could be considered a limitation of the protocol. However, this choice was made to ensure greater ecological validity. The participants’ uncontrolled diet, particularly in terms of carbohydrate and protein intake, is also considered a limitation. Although the study uses a within-subject design, this factor could still restrict training responses. Finally, another limitation of the present protocol concerns the absence of counterbalancing based on upper limb dominance. Although randomization was applied to the selection of the limb, the intervention (TRAD or ECC+), and the order of execution, no specific control was implemented to ensure that either the dominant or nondominant arm would perform a given protocol or initiate the session. This decision aimed to preserve the simplicity and feasibility of the protocol’s practical application. Although lateral dominance may influence muscular performance, such an effect could potentially be minimized if, in this study, 10RM testing reveals similar strength values between participants’ right and left upper limbs. This equivalence would suggest that, at least from an initial functional standpoint, dominance may not have a relevant impact on strength capacity. Nevertheless, the literature indicates that dominance can affect neuromuscular performance due to factors such as motor learning, neural recruitment, and habitual use. Therefore, we acknowledge that subtle dominance-related differences may exist and contribute to variability in training responses. As such, researchers applying this protocol are encouraged to consider including dominance as a covariate or to investigate its influence in future analyses, particularly in studies with greater statistical power.

Studies on strength training frequently use samples predominantly composed of men, which results in a significant bias in the application of findings to the female population. This limitation underscores the need for research that explores the impact of different training protocols specifically on women, enabling the development of evidence-based guidelines tailored to this group. Conducting studies that promote the enhancement of strength and muscle health in women also presents a significant social impact, contributing to the demystification of historical prejudices related to women’s participation in strength training. Additionally, evidence suggests that women exhibit greater fatigue resistance compared to men, which can influence responses to different training intensities and volumes [66]. This superiority may be associated with the higher proportion of type I muscle fibers in women, conferring them greater resistance capacity to repetitive contractions, particularly in muscles such as elbow flexors, as used in protocols like ECC+ [67,68]. Moreover, studies indicate that women tend to recover faster after training sessions, which can directly impact the optimal periodization and training frequency [69].

### Conclusion

This study aims to demonstrate whether the ECC+ protocol, which includes additional eccentric contractions to failure, can be effective in promoting greater adaptations of localized muscle hypertrophy when compared to the TRAD protocol. Both may result in significant morphological changes, such as muscle thickness, mainly in the distal portions. In addition, both may be effective in increasing concentric and eccentric strength, but the ECC+ protocol may maximize overall strength gains. If our hypotheses are confirmed, the results may suggest that additional eccentric training may be an effective strategy to provide strength and hypertrophy gains, especially in specific areas of the muscle. In practice, this means that it may be effective for a trainer to assist in extra eccentric repetitions in order to increase the total work volume of the muscles during a resistance training session to induce better responses in both strength and hypertrophy. Furthermore, it will be another study with a female target audience, which can be used to prescribe exercises for this audience.

## Data Availability

While the experiment is underway, there are currently no datasets to be presented. However, the final datasets will be made available by the corresponding author on reasonable request.

## Abbreviations

DXA: dual-energy X-ray absorptiometry
FAPESP: Fundação de Amparo à Pesquisa do Estado de São Paulo
MFCON: Concentric muscle failure
MFEXC: Eccentric muscle failure
RM: percentage of one-repetition maximum
RPE: Rate of perceived exertion
RT: Resistance training
RTD: Rate of torque development
TRAD: traditional training
VL: volume load
